# 非小细胞肺癌脑转移全脑及病灶局部大分割放射治疗

**DOI:** 10.3779/j.issn.1009-3419.2016.04.08

**Published:** 2016-04-20

**Authors:** 星婷 辜

**Affiliations:** 1 610041 成都，四川大学华西医院肿瘤中心 Cancer Center, West China Hospital, Sichuan University, Chengdu 610041, China; 2 610041 成都，四川大学华西医院肺癌中心 Lung Cancer Center, West China Hospital, Sichuan University, Chengdu 610041, China

**Keywords:** 肺肿瘤, 脑转移, 全脑放疗, 副反应, Lung neoplasms, Brain metastasis, Whole brain radiotherapy, Treatment side effects

## Abstract

高达40%的非小细胞肺癌患者在疾病进程中出现脑转移，且非小细胞肺癌脑转移常为多发转移。脑转移患者的预后较差，中位生存期不到1年。脑转移的放射治疗已经从全脑放疗逐渐发展到多种放射治疗策略广泛应用的时代。目前已证实单纯全脑放疗、手术+全脑放疗、立体定向放射治疗+全脑放射治疗、同步调强全脑放射治疗等对比未治疗患者能提高总生存期。近年来，全脑放疗对认知功能的损害受到广泛关注，针对预期生存时间较长的患者，采取何种放疗模式尚存在争议。本文将分别论述非小细胞肺癌脑转移不同的全脑放射治疗策略及治疗副作用。

## Abstract (en)

Up to 40% non-small cell lung cancer patients developed brain metastasis during progression. Multiple brain metastases are common in non-small cell lung cancer. The prognosis of brain metastasis is poor with median survival of less than 1 year. Radio therapy for brain metastases has gradually developed from whole brain radiotherapy (WBRT) to various radiation strategies. WBRT, surgery+WBRT, stereotactic radiotherapy+WBRT or WBRT with simultaneous integrated boost (SIB), *etc*. have better overall survival than those untreated patients. The damage of the cognitive function from WBRT has been realized recently, however, options of radiation strategies for long expected survival patients remain controversial. This paper will discuss different WBRT strategies and treatment side effects of non-small cell lung cancer with brain metastases.

脑转移瘤是癌症患者常见的并发症，约20%-40%的癌症患者会出现脑转移^[1, 2]^。随着肺癌的发病率逐年升高，非小细胞肺癌（non-small cell lung cancer, NSCLC）脑转移的发病率随之升高，高达30%-50%，40%的NSCLC患者在疾病发展过程中会出现脑转移^[3-6]^，影响中枢神经系统功能^[7]^，并出现与病灶位置、范围相关的神经症状和体征^[8]^。脑转移者不经治疗，平均生存期仅1个月-2个月^[9, 10]^。

## 1 单纯全脑放疗（whole brain radiotherapy, WBRT）

脑转移瘤常伴有明显的瘤周水肿，肾上腺皮质激素的应用，改善了脑转移患者的临床症状和生存质量，中位生存期提高到2个月-3个月。WBRT的价值早在20世纪50年代就有学者报道^[11]^，20世纪70年代WBRT逐渐成为脑转移瘤的标准治疗方案^[12-17]^，WBRT后疾病缓解率约24%-55%^[17-20]^，中位生存期延长至3个月-6个月。自70年代开始就有不少随机试验在探讨WBRT不同的剂量分割方案，2015年美国国家综合癌症网（National Comprehensive Cancer Network, NCCN）指南推荐NSCLC脑转移瘤可接受20 Gy-40 Gy/5次-20 Gy的全脑照射剂量，30 Gy/10次或37.5 Gy/15次为最佳方案，对于一般情况较差的多发脑转移患者可采用20 Gy/5次的照射方式缓解症状。但随访结果显示，WBRT后转移灶局部未控率仍较高，究其原因主要是因为脑组织、晶体、眼球、视神经等正常组织存在剂量（体积）限制，全脑放疗剂量无法达到肿瘤致死剂量。那么能否通过加强肿瘤局部治疗进一步提高治疗效果呢？

## 2 手术联合WBRT

20世纪80年代开始对NSCLC单发脑转移采取手术切除治疗，术后给予放疗，其生存时间（overall survival, OS）较单纯放射治疗延长至10个月^[21, 22]^。外科手术可以迅速减轻占位效应，缓解颅内高压症状。局部手术切除+WBRT也是目前颅内单发脑转移瘤的标准治疗之一^[23]^。Patchell^[24]^将95例单发脑转移瘤患者随机分为手术组和手术+WBRT组，结果提示术后行WBRT明显降低了局部复发率（70% *vs* 18%, *P* < 0.001）及神经相关死亡率（44% *vs* 14%, *P*=0.003），延长了OS（15周*vs* 40周，*P* < 0.01）和生活自理时间（8周*vs* 38周，*P* < 0.05）。但大约只有30%患者适合行外科治疗，多发转移瘤、一般情况较差、或合并有严重并发症患者并不适合或不能耐受外科治疗^[25]^。

## 3 立体定向放射治疗（stereotactic radiotherapy, SRT）联合WBRT

近20年来，依赖于影像学技术的发展，SRT在脑转移瘤治疗中的价值得以体现。SRT是一种“高精度”的放射治疗技术，利用影像辅助，给予靶区高剂量（4 Gy-25 Gy）放射治疗，同时可最小化周围健康组织的损害。通过SRT技术可以给予病灶局部较高剂量达到更好治疗效果，且避免周围正常脑组织的损害。和外科手术相比，SRT微创，无手术相关死亡，水肿及放射性坏死等晚期并发症少见。

### 3.1 立体定向外科治疗（stereotacticradiosurgery, SRS）+WBRT

SRS后是否需要联合WBRT，目前尚无定论。WBRT联合SRS治疗有降低颅内复发率及颅内远处转移率等方面的优势^[26-29]^，部分研究^[30, 31]^认为联合WBRT能延长生存期。在Pirzkall^[30]^的研究中SRS联合WBRT可将单纯SRS的中位生存期从8.3个月延长到15.4个月，尤其对于不伴有颅外病灶的患者。但几项大型前瞻性研究提示联合WBRT在延长OS无获益^[26-28, 32]^。JROSG99-1研究^[26]^将直径小于3 cm、病灶数1个-4个的132例脑转移瘤患者随机分为SRS+WBRT（65例）和SRS组（67例）两组，中位生存期（8个月*vs* 7.5个月）、1年生存率、放射性不良反应均无统计学差异，但行WBRT的患者颅内病灶控制率显著高于未行WBRT的患者，1年局部复发率分别为46.8%、76.4%（*P* < 0.01）。EORTC22952-26001^[27, 28]^研究入组了359例1个-3个脑转移瘤的患者，手术或者SRT治疗后根据是否行WBRT将患者分为观察组及WBRT组。结果提示WBRT未能改善OS及神经功能，但降低了2年颅内复发率（78%: 48%, *P* < 0.001）。

美国神经外科医师联合会和神经外科医师大会（建议新诊断的、最大直径小于3 cm、占位效应轻（中线移位小于1 cm）的成人实性脑转移瘤可行SRS+WBRT^[33]^。NCCN指南推荐对于1个-3个脑转移灶可手术患者行手术序贯WBRT（1类推荐），或者SRS，单发脑转移瘤患者行SRT+WBRT（1类推荐），选择开颅手术还是SRS取决于肿瘤大小和位置。有经验的治疗团队治疗位置深在的小型脑转移瘤，SRS治疗往往能取得不错的治疗效果。

### 3.2 分次立体定向放射治疗（fractionated stereotactic radiotherapy, FSRT）+WBRT

NCCN指南推荐：根据肿瘤大小（直径≤20 mm，21 mm-30 mm，31 mm-40 mm），可耐受的最大剂量分别为24 Gy、18 Gy、15 Gy^[34, 35]^。RTOG90-05研究指出对于颅内单发肿瘤且直径大于3 cm者，SRT最大耐受剂量为15 Gy/次，18 Gy/次将产生严重的3级-5级神经毒性。此时，采用FSRT可能是更好的选择，即采用3次-5次的照射，每次剂量为7 Gy-8 Gy（最多不超过11 Gy-12 Gy），总体等效剂量为SRS的70%-80%。与SRT相比，FSRT具有以下优势：①分次剂量较SRS低，有利于保护肿瘤周围的重要结构和器官（如脑干、视通路），尤其适合于治疗脑中线部位病灶；②FSRT更适合治疗较大体积病灶，可减少肿瘤周围正常组织的损伤。一项Ⅱ期临床研究^[36]^评价了FSRT治疗脑转移的疗效及毒性反应，51例患者中有72个脑转移灶，分别采取5×6 Gy或5×7 Gy的方式进行放疗，中位随访7个月，结果提示完全缓解（complete response, CR）、部分缓解（partial response, PR）、疾病稳定（stable disease, SD）、疾病进展（progressive disease, PD）分别为66.7%、18.1%、12.5%、2.8%，中位生存期11个月。副反应与正常组织受照剂量相关，V4 Gy≥23 cm^3^的患者多数出现明显的放射反应在魏微等^[31]^的研究中，采用FSRT的患者主要为位于中线部位的病灶（7/7）和直径 > 3 cm的较大病灶（25/32），其疗效与SRS相当，且无严重的急性反应，提示采用FSRT方式治疗中线部位和较大体积的脑转移病灶是有效和安全的。另一项针对1个-3个脑转移灶的Ⅱ期临床研究^[37]^中，40例患者采用FSRT，神经相关死亡率为13%（5例，包括3例不明原因死亡者），中位总生存期（median survival time, MST）、无进展生存期（progression-free survival, PFS）分别为16个月（9个月-23个月）、11个月（4个月-21个月），其治疗安全性、疗效不亚于SRS。意大利学者^[38]^分析了47例1个-2个脑转移灶患者在WBRT治疗（中位剂量37.50 Gy）后分别采用FSRT和SRS治疗，17例SRS患者中位治疗剂量15 Gy，30例FSRT患者中位治疗剂量20 Gy，中位随访时间102个月（17个月-151个月）。在SRS和FSRT中，MST分别为22个月、16个月（*P*=0.4），1年生存率分别为56%、62.1%，5年生存率分别为16%、3%，1年局控率分别为80%、61.1%（*P*=0.15）。提示在WBRT治疗后不管是SRS还是FSRT疗效无明显差异。有文献报道，对于较大的脑转移瘤（直径 > 3 cm），SRT一年局控率分别为37%-62%，FSRT则为70%以上^[35, 39-42]^，对于较大体积的脑转移瘤，FSRT可作为治疗方式之一。

## 4 WBRT同步调强（whole-brain radiotherapy with simultaneous integrated boost, WBRT-SIB）

近年来，随着调强放疗技术的不断发展，WBRT-SIB也可作为脑转移病灶的放射治疗选择。周麟^[43]^回顾了87例接受WBRT（40 Gy/20 f）+同步图形引导下调强适形放疗（20 Gy/5 f）（image guided intensity-modulated radiotherapy, IG-IMRT）的NSCLC脑转移患者，1年颅内控制率、局部未控率、远处脑组织未控率分别为62.9%、13.8%、19.2%，2年为42.5%、30.9%、36.4%。mPFS及MST均为10个月。在Kim等^[44]^的研究中，11例肺腺癌脑转移患者（共70个转移灶）接受了WBRT-SIB治疗，中位随访时间14个月（3个月-25个月），1年颅内转移灶控制率为67%，无3级以上毒性反应。目前WBRT同步调强瘤床推量能否广泛应用于NSCLC脑转移患者的治疗中，仍需相关大型前瞻性临床试验数据予以证实。

## 5 WBRT的副作用

2014年美国放射肿瘤学会（American Society for Radiation Oncology, ASTRO）会议建议：不要将WBRT常规加入到SRS对局限脑转移的治疗中。随机试验已证实WBRT+SRS对比SRS无生存获益，且会损伤患者的认知功能、增加疲惫感、降低生活质量。针对预期生存时间较长的患者，WBRT易引起迟发性脑白质病（delayed leukoencephalopathy, DLE），导致患者痴呆、认知功能障碍、尿失禁、易怒等后遗症，极大的降低了患者的生存质量^[45]^。有学者^[46]^对比了WBRT+SRS及SRS患者的MRI，发现生存时间超过1年的患者中，仅接受SRS患者的DLE发生率明显低于WBRT+SRS组（97.3% *vs* 3.2%, *P* < 0.001）。MD. Anderson肿瘤中心开展的一项前瞻性随机试验^[47]^评估了单发转移瘤患者接受SRS+WBRT的神经认知功能，对比SRS，1年局控率分别为100% *vs* 67%，1年脑复发率分别为27% *vs* 73%（*P*=0.000, 3），但接受了WBRT的患者在4个月时的学习、记忆功能发生减退的可能性为96%。

海马体及边缘系统在记忆形成中的重要作用已被逐渐认识，避免海马区域的全脑放疗^[48, 49]^，及相关神经类药物^[50, 51]^已应用在临床试验中，且获得一定进展。

## 6 预后因素

影响NSCLC脑转移瘤预后的非治疗因素很多，多篇文献指出患者年龄、KPS评分、原发灶控制、颅外有否转移、转移灶是否多发等对预后有显著影响^[52-56]^。上世纪90年代开始使用递归分区分析（recursive partitioning analysis classes, RPA）评估脑转移瘤预后，随后等级预后评估标准（graded prognostic assessment index, GPA）、脑转移基本评分（basic score for brain metastases, BSBM）等被应用于临床。肺内原发病灶的分期越早、KPS评分越高，脑转移患者的生存率越高，对于这类患者，在处理颅内转移灶的同时，肺内原发病灶也需要积极处理。放疗医师应全面评估患者的年龄、KPS评分、转移灶位置、大小、数目、肺内原发病灶情况，根据预后评估模型判断患者预后，与患者充分沟通后，选择适合的治疗方式。

## 7 结论

综上所述，WBRT作为NSCLC脑转移瘤的标准治疗方案已有近半个世纪；对于单发转移瘤，手术联合WBRT显示出了明显的生存获益；SRT联合WBRT在降低颅内复发率和颅内远处转移率等方面的优势有目共睹，但其对比单纯SRS并无生存获益，且对患者的认知功能损伤较大。在关注放疗效果的同时，如何减轻治疗副反应，避免认知功能的损伤，是NSCLC脑转移放疗中需要关注的焦点。
